# The current status of Charcot-Marie-Tooth disease type 1 A treatment

**DOI:** 10.1007/s13760-025-02881-1

**Published:** 2025-08-27

**Authors:** Hongdan Qi, Xin Wang, Bing Wu, Jing Chen, Gang Zhang

**Affiliations:** Department of Neurology, Children’s Hospital of Nanjing Medical University, Nanjing, 210019 China

**Keywords:** Charcot-Marie-Tooth disease, CMT1A, *PMP22*, Gene therapy, Stem cell therapy, Hereditary peripheral neuropathy

## Abstract

Charcot-Marie-Tooth disease type 1 A (CMT1A) is the major subtype of hereditary peripheral neuropathies and arises from a 1.5 megabase (Mb) tandem duplication in chromosome 17p11.2-p12 that contains the complete peripheral myelin protein 22 (*PMP22*) gene. Patients commonly present with progressive weakness and atrophy of the distal muscles, accompanied by hyperalgesia, decreased or absent tendon reflexes, and foot deformities. Current clinical management relies on multidisciplinary supportive care. Recent preclinical studies targeting potential therapeutic strategies for CMT1A have focused on correcting the gene-dose imbalance of *PMP22*. Notably, PXT3003 has shown phase III clinical efficacy in relieving symptoms and reducing neuropathy, and is expected to be the earliest CMT1A-targeted drug on the market. Gene editing approaches have also shown therapeutic promise in animal models, but off-target effects remain a concern. In addition, the rapid development of induced pluripotent stem cell (iPSC) technology has paved the way for stem cell therapies, which may be a promising therapeutic approach. This article reviews the existing literature on therapeutic strategies for CMT1A and aims to provide a valuable reference for the clinical treatment of CMT1A.

**Supplementary Information:**

The online version contains supplementary material available at 10.1007/s13760-025-02881-1.

## Introduction

CMT is a highly heterogeneous group of inherited peripheral neuropathies with an overall prevalence of approximately 1/2500 [1]. CMT1A accounts for approximately 50% of all diagnosed cases of CMT. The clinical features include progressive weakness and atrophy of the distal muscles, often accompanied by decreased sensation, decreased or absent tendon reflexes, and foot deformities. It is caused by a tandem duplication of approximately 1.5 Mb in the chromosome 17p11.2-p12 region, which contains the complete *PMP22* gene, whereas point mutations in the *PMP22* gene itself are only seen in a very small number of atypical cases [40, 41]. This genetic variant makes Schwann cells dysfunctional through overexpression of *PMP22*, leading to poor myelination and axonal degeneration [2].

To date, there is no effective treatment for CMT1A, and current clinical management remains symptomatic supportive therapy, including surgical orthotics, rehabilitation and surgery. With the development of second-generation gene sequencing (NGS) technology and in-depth research on pathogenesis, a number of targeted therapies for CMT1A are undergoing preclinical and clinical evaluation, mainly including targeted drug therapy, gene silencing therapy and stem cell therapy, whose main mechanism is to correct the gene dosage imbalance of *PMP22.*

In this article, we will focus on the available treatments and the latest research advances for CMT1A to provide clinicians with the most up-to-date clinical guidance.

## Literature search methodology

To ensure a comprehensive assessment of therapeutic advances in CMT1A, this review synthesised evidence retrieved from PubMed, Web of Science, using keywords including “Charcot-Marie-Tooth”, “CMT1A” and “gene therapy”, focusing on original studies, clinical trials and authoritative reviews, while excluding non-peer-reviewed studies. Interventional studies were screened on ClinicalTrials.gov, WHO ICTRP, and EU-CTR, including phase III trials of PXT3003 (NCT04762758) and early gene therapy trials (e.g., AAV-shRNA). The inclusion of high-impact reviews and original research, provides a reliable basis for the molecular genetic mechanisms and therapeutic innovations in this paper and ensures the latest preclinical and clinical advances.

## Symptomatic supportive treatment for CMT1A

### Surgical orthopedic therapy

As CMT1A is characterized by the development of tendon contractures, foot deformities and foot drop, treatment with ankle-foot orthoses (AFOs) can improve the quality of life of patients to a certain extent. A controlled study by Menotti et al. included a group of patients with CMT1A to evaluate the effect of an anterior elastic AFOs on patients’ walking economy. The results showed that anterior elastic AFOs can provide support, improve patient gait, and increase walking tolerance [3].

However, the selection of orthoses should ideally be individualized and reassessed periodically during customization and use to ensure that the device remains suitable for the current stage of the disease.

### Rehabilitation therapy

There is no specific treatment for CMT1A, so comprehensive rehabilitation is crucial for CMT1A patients. Since muscle atrophy and sensory deficits are the main characteristics of CMT1A patients, rehabilitation therapy aims to improve both motor and sensory deficits.

A study of 8 patients with CMT who participated in a 6-month cardiorespiratory and proprioceptive rehabilitation program showed an increase in ankle mobility and a lengthening of the 6-minute walk test stride distance in all participants [6]. A clinical trial by El Mhandi et al. of a 24-week intermittent cycling training program for patients with CMT also demonstrated that intermittent aerobic exercise improved cardiorespiratory function, muscle strength and daily motor function in patients with CMT [5].

Although the above studies were not patient specific to the CMT1A, they are still informative. It is reasonable to assume that comprehensive rehabilitation, especially exercise therapy, plays an important role in improving motor function, preventing and delaying disease progression.

### Surgical therapy

For foot deformity in patients with CMT1A, most patients tend to choose conservative treatment to improve the deformity, but some patients choose surgical treatment due to the ineffectiveness of conservative treatment or the serious impact on their lives, especially in advanced stages [7]. The study by Ferraro et al. included 5 patients with CMT1A who underwent functional surgery and underwent 3 weeks of intensive neurorehabilitation after removal of the cast showing a reduction in fatigue, pain, and other symptoms and an overall improvement in gait and balance, demonstrating that the combination of functional surgery and early intensive neurorehabilitation may be an effective approach to improving clinical symptoms in patients with CMT1A [8].

The above study can indicate that surgery may be an effective treatment for CMT1A, which can effectively correct foot deformity and improve pain, and the combination of postoperative rehabilitation training can achieve the best therapeutic effect.

## Targeted treatment for CMT1A

### CMT1A treatment with compounds and drugs

Preclinical studies have emphasized the importance of *PMP22* dosage in the pathogenesis of CMT1A, and therefore pharmacological treatment of CMT1A has focused on reducing the expression of this gene and supporting effective myelin formation. However, most drug therapies do not specifically reduce *PMP22* gene expression, so long-term efficacy and safety remain to be addressed.

#### Ascorbic acid

Ascorbic acid was one of the first therapies to evaluate the efficacy of CMT1A treatment. In vivo studies have shown that ascorbic acid inhibits *PMP22* expression and promotes myelin formation and improved motor function [9]. Jennings MJ et al. included 34 randomized controlled trials on drug therapy in patients with CMT1A, and an analysis of this data found no therapeutic benefit of ascorbic acid in CMT1A [37].

#### PXT3003

PXT3003 is an oral liquid with a mechanism of action that may be to modulate the cAMP pathway to inhibit the transcription of *PMP22* or to improve the folding of the Pmp22 protein in order to promote the differentiation of Schwann cells and to promote myelin formation. A phase II clinical trial (NCT01401257) enrolling 80 adult patients with CMT1A demonstrated that PXT3003 had a favorable safety and tolerability profile and significantly improved neuropathy in the highest dose group compared to the other groups. The pivotal Phase III trial (NCT04762758) enrolled 350 adults with CMT1A and completed its 15-month double-blind phase in October 2023. Results demonstrated significant improvements in exercise capacity and neuropathy severity in the PXT3003 group compared to placebo. Furthermore, an open-label extension phase is ongoing to evaluate long-term safety until regulatory approval. Additionally, a Phase III trial (CTR20211722) was completed in March 2024 for China. Based on these data, in April 2025, the Tianshili Group submitted a New Drug Application (NDA) to the Center for Drug Evaluation (CDE) in China. These findings highlight that PXT3003 has therapeutic potential for patients with CMT1A.

#### Progesterone receptor antagonists

Progesterone is a neuroactive steroid that indirectly stimulates the promoters of myelin-related genes, including the *PMP22* gene, in the Schwann cell, while its derivatives also induce myelin gene expression by activating GABAA receptors present in the Schwann cell. Subcutaneous injection of the progesterone receptor antagonist onapristone in the CMT1A rat model significantly ameliorated behavioral abnormalities by decreasing *PMP22* levels and improving axonal structure [2]. Currently, clinical trials have not been applied to patients with CMT1A due to the many adverse effects of onapristone, so the potential of onapristone for the treatment of CMT1A remains uncertain.

#### Neuregulin type 1-III

Peripheral nerve myelin formation is supported by activation of axonal neuromodulin type 1-III (NRG1-III), and myelin thickness is controlled by the amount of NRG1-III. Under conditions of NRG1 dysfunction, neuronal degeneration and abnormal myelin thickness are observed. It was found that short-term intraperitoneal administration of rhNRG-1 to CMT1A rats at 6–18 days after birth promoted Schwann cell differentiation [11]. However, another study found that the expression of soluble NRG1 increased significantly after 16 days of life in CMT1A rats, so it is hypothesized that the activity of the NRG1/ErbB pathway may be related to the individual’s developmental stage [12]. Thus, modulation of NRG1-III levels is a potential treatment, but individualized treatment regimens should be chosen for patients of different ages.

#### Sephin-1

Sephin-1, an inhibitor of selective phosphatase 1 regulatory subunit 15 A (PPPIR15A), inhibits eIF2α dephosphorylation and prolongs the reduction of protein translation in response to stress, reducing endoplasmic reticulum stress-induced cell death. Sephin-1 is currently being evaluated in the CMT1A rodent model, making it a potential candidate for clinical trials in CMT1A and CMT1B [13].

#### ACE-083

ACE-083 inhibits muscle atrophy by binding to specific proteins in the TGF-β protein superfamily that inhibit muscle growth, making it particularly indicated for neuromuscular disorders where specific muscle atrophy is severe. Preclinical studies demonstrated that ACE-083 improved isometric contractility of the tibialis anterior muscle and ankle dorsiflexion torque in CMT mice, suggesting its potential efficacy in treating patients with CMT1A [14].

#### Curcumin

Curcumin is an antioxidant compound [15]. An in vitro study found that oral supplementation with curcumin abrogated the unfolded protein response in the endoplasmic reticulum and alleviated locomotor deficits in Tr-J mice in a dose-dependent manner with low toxicity even at high doses [16]. To overcome the unstable pharmacokinetics of curcumin, researchers developed curcumin-rich cyclodextrin/cellulose nanocrystals (NanoCur). In vitro and in vivo experiments in the CMT1A rat model revealed a significant increase in nerve conduction velocity, sensory-motor coordination and neuromuscular strength in CMT1A rats using NanoCur, as well as histologic findings that NanoCur was able to reestablish an almost normal myelin phenotype [17]. The above studies validate curcumin as a therapeutic approach to improve nerve damage in CMT1A.

#### Melatonin

Melatonin is another antioxidant with anti-inflammatory properties used to treat cellular stress caused by *PMP22* overexpression in CMT1A. Supplements containing melatonin have been approved by the FDA (Food and Drug Administration) and are widely used in clinical studies. A study found that children with CMT1A treated with melatonin had reduced serum oxidative markers and balanced levels of glutathione circulating and pro-inflammatory cytokines. This suggests that melatonin may have a therapeutic effect in patients with CMT1A by attenuating peroxidative and inflammatory conditions and decreasing degenerative processes, but whether it is beneficial for the neuropathology of CMT1A remains to be further confirmed [18].

#### Lipid supplementation

Dietary lipid supplementation may promote cholesterol and phospholipid biosynthesis by directly compensating for lipid metabolism defects in Schwann cells [19]. Supplementation of phosphatidylcholine and phosphatidylethanolamine in the CMT1A rat model resulted in significant improvements in motor function, muscle volume and strength, and an increase in the number of myelinated axons [19]. Current clinical trials are unclear whether high doses of dietary phospholipids would benefit CMT1A patients [2]. Lipid diet therapy may become an important strategy due to no significant side effects, low treatment cost, safety, tolerability, and ease of clinical trials.

#### Dietary restrictions and Rapamycin

Functional benefits of dietary restriction include enhanced myelin expression, increased myelin thickness, and downregulation of abnormal Schwann cell proliferation. Subjecting the Tr-J mouse model of CMT1A to an intermittent fasting regimen revealed decreased *PMP22* expression, and improved myelin formation and motor performance [20]. Intermittent fasting is not clinically translatable, so studies introducing the dietary restriction mimetic rapamycin into ex vivo cultures of C22 mice found that rapamycin improved the processing of Pmp22 protein and increased the contouring of myelin interneurons, as well as increasing the production of other myelin-related proteins [21]. However, rapamycin failed to improve neuromuscular performance in vivo in the Tr-J model.

#### Histone deacetylase 6 inhibitor

The histone deacetylase 6 (HDAC6) Inhibitor CKD-504 regulates the acetylation of nuclear and cytoplasmic proteins, including heat shock protein 90 (HSP90) and 70 (HSP70), which are involved in the folding/refolding of proteins including Pmp22 [22]. A study demonstrated that CKD-504 enhanced HSP90 acetylation and HSP70 expression and reduced Pmp22 protein levels in MSC-derived Schwann cells from CMT1A patients and in a C22 mouse model of CMT1A, confirming behavioral, electrophysiological, and histological improvements [22]. Thus, the novel HDAC6 inhibitor CKD-504 may be therapeutically effective in CMT1A. (Table. 1).

**Table 1:** Overview of treatments with compounds and drugs

Treatment	Mechanism	Reference
Ascorbic Acid	Blocks adenylate cyclase activity to inhibit *PMP22* expression	[9]
PXT3003	Regulates the cAMP pathway to inhibit *PMP22* expression or improve Pmp22 protein folding	[23, 24]
Progesterone Receptor Antagonists	Inhibits progesterone receptor activity and expression of myelin-related genes	[2]
NRG1-III	Promotes Schwann cell differentiation and regulates myelin thickness	[11]
Sephin-1	Inhibits eIF2α dephosphorylation to reduce endoplasmic reticulum stress-induced apoptosis	[13, 23]
ACE-083	Binds to and inhibits select proteins in the TGF-β protein superfamily that inhibit muscle growth	[14]
Curcumin	Abrogates the unfolded protein response activated by Pmp22 protein retention in the endoplasmic reticulum	[15]
Melatonin	Reduces peroxidative and inflammatory conditions	[18]
Lipid Supplementation	Promotes cholesterol and phospholipid biosynthesis by directly compensating for lipid metabolism defects in Schwann cells	[19]
Dietary Restrictions and Rapamycin	Enhances myelin expression, increases myelin thickness and downregulates abnormal Schwann cells	[20, 21]
HDAC6 Inhibitor	Regulates acetylation of nuclear and cytoplasmic proteins	[22]

Note: PXT3003, an oral liquid combination of baclofen, naloxone and sorbitol; cAMP, cyclic adenosine monophosphate; NRG1-III, Neuregulin type 1-III; eIF2α, eukaryotic translation initiation factor 2α kinase; ACE-083, a locally functional follicle inhibitor-based fusion protein; TGF-β, transforming growth factor β; HDAC6 histone deacetylase 6. This table was adapted with some changes from Stavrou M et al. Int J Mol Sci. 2021, 22: 6048 [2]

### CMT1A treatment with Gene-Mediated therapy

CMT1A is caused by a tandem repeat of approximately 1.5 Mb located on the region of chromosome 17p11.2-p12 that contains the complete *PMP22* gene [40, 41]. The pathogenesis of CMT1A remains unclear, but it has been suggested that its pathogenesis may be related to the overexpression of *PMP22* and interference with the proteasomal degradation pathway. The accumulation of Pmp22 protein in the perinuclear and cytoplasmic compartments, coupled with a generalized decrease in proteasome activity, leads to the accumulation of unfolded proteins, which in turn causes endoplasmic reticulum stress [23, 24]. Furthermore, accumulation of unfolded proteins activates the unfolded protein response in Schwann cells, leading to apoptosis, impaired myelin function, and secondary axonal degeneration, ultimately leading to neurological damage [23, 24]. Therefore, reducing the overexpression of the *PMP22* gene at the DNA or mRNA level may become an effective method for the treatment of CMT1A. Of particular note, excessive inhibition of *PMP22* may lead to another inherited peripheral neuropathy, hereditary neuropathy with liability to pressure palsies (HNPP) [42]. This opposing gene dosage effect highlights the importance of maintaining precise regulation of *PMP22* expression levels. Current gene therapy strategies rely on precise delivery platforms, including viral vectors (e.g., adeno-associated viruses, lentiviruses, and virus-like particles) and non-viral vectors (e.g., lipid nanoparticles and exosomal delivery), which are the technological basis for RNA interference, antisense oligonucleotides, and CRISPR/Cas9 therapies aimed at restoring *PMP22* homeostasis [27]. (Table. 2).

**Table 2:** Overview of treatments with Gene-Mediated therapy

NT-3	Promotes Schwann cell regeneration and myelin formation	[25]
siRNA	Selectively reduces the expression level of the *PMP22* gene	[26]
shRNA	Selectively reduces the expression level of the *PMP22* gene	[27]
miRNA	Selectively reduces the expression level of the *PMP22* gene	[28–30]
P2 × 7 Receptor Antagonist	Reduces the expression level of the *PMP22* gene	[23, 24]
ASOs	Promotes degradation of targeted mRNAs	[32]
ATFOs	Binds to the *PMP22* promoter to repress transcription	[24]
CRISPR/Cas9	Manipulates the regulatory elements in the *PMP22* gene to reduce transcription	[33, 38]
Hepatocyte Growth Factor	Stimulates Schwann cell repair and promotes peripheral nerve regeneration	[23, 24]

Note: NT-3, neurotrophin 3; siRNA, small interfering RNA; shRNA, short hairpin RNA; miRNA, microRNA; ASOs, antisense Oligonucleotides; ATFOs, antiparallel trimer-forming oligonucleotides; This table was adapted with some changes from Stavrou M et al. Int J Mol Sci. 2021, 22: 6048 [2]

#### Gene silencing therapy

##### RNA interference (RNAi)

RNA interference (RNAi) has become one of the main targets for CMT1A gene therapy. RNAi is a technique involving small interfering RNAs (siRNAs), short hairpin RNAs (shRNAs), and natural and artificial microRNAs (miRNAs). Mature allele-specific siRNAs, shRNAs, and miRNAs are concatenated with RNA-induced silencing complexes (RISCs) in the cytoplasm and bind complementarily to targeted *PMP22* mRNA sequences, thereby selectively reducing the expression level of the *PMP22* gene. After intraperitoneal injections of *PMP22*-targeted siRNA every three days for a total of five injections in the CMT1A mouse model, improvements in myelin sheath contour, muscle volume, motor function, and electrophysiological performance were found within 3 weeks of the last administration [26]. To achieve a more durable RNA interference treatment, intrathecal injection of *PMP22*-targeting shRNA into the CMT1A rat model after packaging into the AAV2/9 vector allowed for long-term RNAi treatment. The study found that more than 70% of Schwann cells were transduced, improved myelin formation, and persisted for up to 12 months after injection. However, this approach may be more difficult to translate due to the risk of fiber damage and the need for focused anesthesia [26, 27]. In addition, some researchers injected lentiviral vectors expressing natural miR-318 into the nerves of C22 mice and found that miR-318 targeted the 3’UTR of the *PMP22* gene, resulting in silencing of the *PMP22* mRNA and protein levels [28]. Another AAV2-mediated miR-29a binds to a conserved site in the *PMP22* gene and negatively regulates its translation, reducing *PMP22* mRNA and protein levels [29]. Recently newly developed artificial miR-871 packaged into an AAV9 vector and delivered into adult C61het mice, AAV9-miR 871 targeted the 3 ‘-UTR of exon 5 of human *PMP22* and murine *PMP22*, silencing about 50% of the Schwann cells by more than 50% [30]. Notably, overexpression of natural or artificial miRs may compete with and dysregulate normal miR biosynthesis, leading to adverse effects on transcriptional regulation and cell signaling [16].

##### Antisense oligonucleotides (ASOs)

Antisense oligonucleotides (ASOs) are single-stranded nucleic acids that specifically bind to *PMP22*-targeted mRNA sequences and block RISC, facilitating degradation of target mRNAs in an RHaseH-dependent manner. Subcutaneous administration of ASO targeting *PMP22* in C22 mice and CMT1A rats reduced mRNA levels of human and murine *PMP22* and improved the restoration of myelination, motor nerve conduction velocities, and compound muscle action potentials to those of wild-type animals in a dose-dependent manner in the CMT1A rodent model [32]. Antiparallel trimer-forming oligonucleotides (ATFOs) are designed to bind to the *PMP22* promoter and compete with transcription factors to bind the cis-regulatory region of DNA to repress transcription [10], but so far none of the sequences have been tested in the CMT1A model.

##### CRISPR/Cas9

The CRISPR/Cas9 approach aims to manipulate regulatory elements in the *PMP22* gene to reduce transcription. CRISPR/Cas9 was used in a rat Schwann cell line in order to deletion of the upstream region of the *PMP22* gene, the deletion of which resulted in reduced levels of *PMP22* mRNA [38]. Similarly, CRISPR/Cas9-mediated deletion of the TATA box promoter of the *PMP22* gene in C22 mice using nonviral intrathecal injection also downregulated *PMP22* mRNA and improved neuropathology [4]. Despite these promising studies, the off-target effects of gene editing approaches remain a concern, and with in vivo gene editing efficacy in injected tissues remaining relatively low [33].

#### The growth factors neurotrophin 3

Overexpression of growth factors neurotrophin 3 (NT-3) in the cytoplasm promotes Schwann cell regeneration and myelin formation [25]. In a preliminary study that included 8 patients with CMT1A, increased myelinated fiber density, neurological deficits, and improved sensory scores were found in all subjects after 6 consecutive months of subcutaneous injections of NT-3. In an effort to develop a one-time treatment, an investigator packaged NT-3 cDNA into an AAV-1 vector and then injected it intramuscularly into a CMT1A mouse model, which showed improved myelin fiber density, function, and electrophysiological performance for up to 48 weeks post-injection, and was well-tolerated for up to 48 weeks without any toxicity issues [25]. Therefore, an additional study was designed to administer incremental doses of the AAV1-NT-3 gene intramuscularly in the legs of CMT1A subjects in a Phase I/IIa clinical trial, which was subsequently suspended due to vector production issues. However, the results have not yet been published.

#### P2 × 7 receptor antagonist

P2 × 7 receptors are essential for neuronal synaptic transmission and interact dose-dependently with overexpression of *PMP22*. *PMP22* overexpression leads to overactivation of P2 × 7 receptors, resulting in an increase in the influx of extracellular Ca^2+^ and Na^+^ into the Schwann cell, which in turn leads to disorganization of Schwann cells [23]. Sociali et al. tested the therapeutic potential of the P2 × 7 antagonist A438079 in CMT1A rats. After treatment with A438079, CMT1A rats showed improved hindlimb muscle strength, electrophysiological and morphological characteristics, and Schwann cell differentiation. Preclinical testing of P2 × 7 antagonists has shown satisfactory safety and tolerability [31].

#### Hepatocyte growth factor

**Fig. 1:**
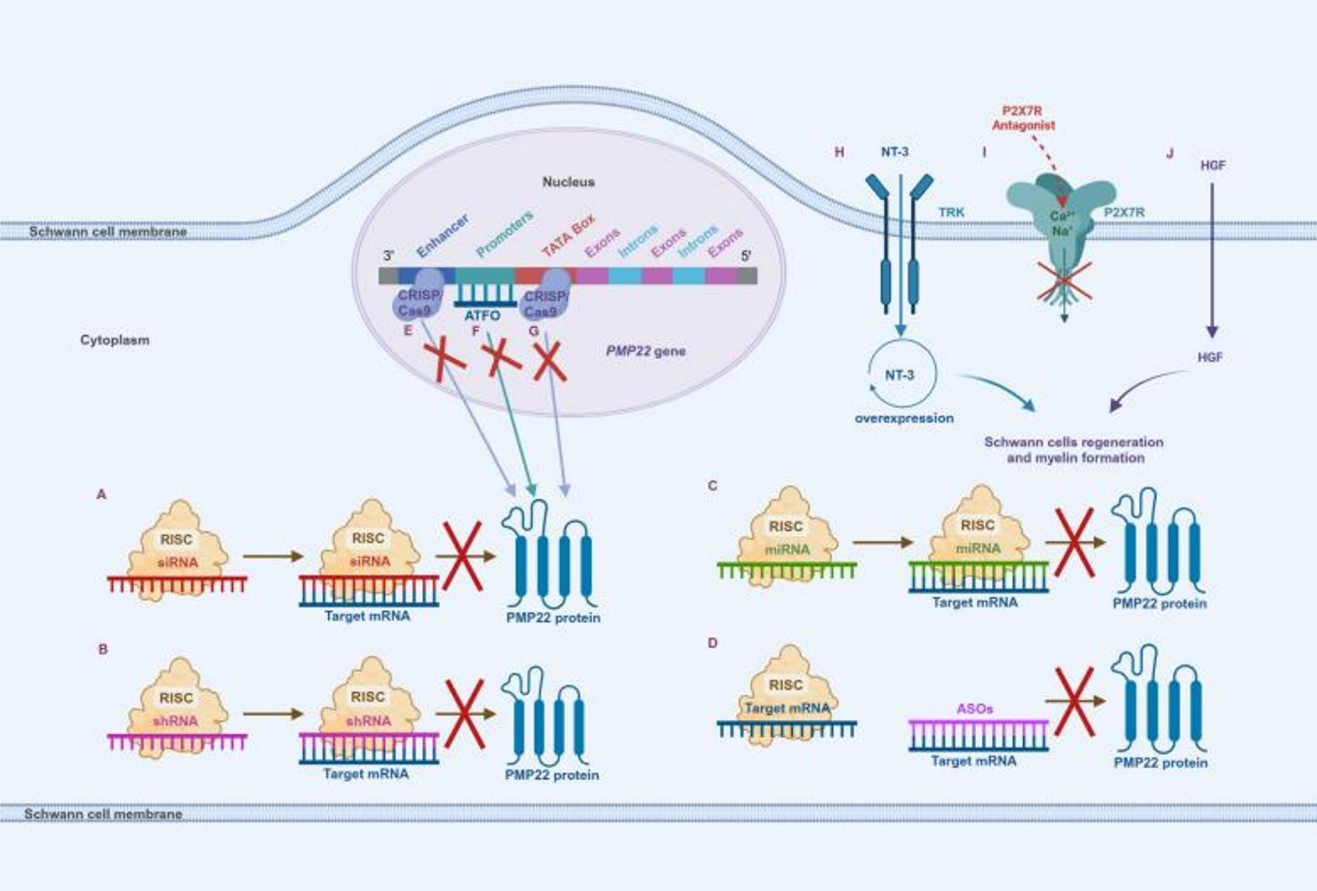
Simple diagram of CMT1A gene therapy [1, 2, 24]. **A-C** Small interfering RNAs (siRNAs; A), short hairpin RNA (shRNA; B), and microRNA (miRNA; C) are concatenated with RNA-induced silencing complexes (RISCs) in the cytoplasm and bind complementarily to targeted *PMP22* mRNA sequences, thereby selectively reducing the expression level of the *PMP22* gene. **D** Antisense oligonucleotides (ASOs) bind specifically to *PMP22* target mRNA sequences and block RISC to promote degradation of target mRNAs. **E-G** CRISPR/Cas9 binds to an enhancer (E) or TATA box (G) upstream of the *PMP22* gene to reduce transcription. Antiparallel trimer-forming oligonucleotides (ATFOs; F) binds to the promoter of *PMP22* to repress transcription. **H-J** Overexpression of the growth factors neurotrophin 3 (NT-3; H) and hepatocyte growth factor (HGF; J) in the cytoplasm promotes Schwann cell regeneration and myelin formation. NT-3 enters Schwann cells via tyrosine kinase receptor type 3. Hepatocyte growth factor enters Schwann cells through the lipid bilayer. P2 × 7 receptor antagonist (I) block aberrant Ca^2+^ and Na^+^ inward flow to reduce *PMP22* expression

VM202 is a non-viral vector encapsulating a novel genomic cDNA heterodimer of human hepatocyte growth factor (HGF). This heterodimer promotes peripheral nerve regeneration by stimulating Schwann cell repair. The FDA granted VM202 orphan drug status in 2014 and classified it as a fast track drug in 2016. A Phase I/IIa clinical trial investigating repetitive intramuscular injections of VMV202 in both legs of CMT1A subjects has been completed. Although the results of the study have not yet been published, repeated intramuscular injections of the vector in patients with ischemic heart disease and amyotrophic lateral sclerosis showed a decline in the beneficial effects of VM202 after a few months, suggesting that this may be a transient symptomatic treatment [24]. (Figure. 1).

### CMT1A treatment with stem cell replacement therapy

Stem cells are a class of cells with self-renewal and multidirectional differentiation that have many potential functions. Stem cell replacement therapy (SCRT) involves the introduction or implantation of healthy stem cells into the body to reduce inflammation and modulate the immune system to repair or replace damaged cells in the body, leading to a cure. In recent years, with the development of stem cell technology, stem cell therapy has brought new hope for neurological diseases, bone diseases, cardiovascular and cerebrovascular diseases and autoimmune diseases.

Recent advances in iPSC-based disease modeling have significantly enhanced our understanding of CMT1A pathogenesis. Van Lent et al. (2023) established the first CMT1A patient-iPSC derived organoid model demonstrating that Schwann cells recapitulated key pathological features consistent with peripheral nerve biopsies from CMT1A patients [39]. Based on this model, a Japanese study used a gene editing approach to target *PMP22* overexpression. Using AAV-mediated delivery of gRNA3 into iPSC-derived Schwann cells from CMT1A patients, it was found that *PMP22* gene duplications were reduced by 20–40%, mRNA and protein expression levels were normalised, and apoptosis and myelination defects were rescued [34]. These results align with Van Lent et al.’s findings that downregulating *PMP22* restores myelin integrity in CMT1A organoids. Together, these studies highlight the therapeutic potential of *PMP22* reduction strategies and validate iPSC-derived models as robust platforms for preclinical testing.

## Conclusion and future perspective

After nearly 30 years of molecular genetics development, CMT has entered the emerging era of molecular targeted therapy from the initial supportive therapy. The etiology of CMT1A is a tandem duplication of approximately 1.5 Mb in the region of chromosome 17p11.2-p12 containing the *PMP22* gene, and its pathogenesis may be dominated by the dosage effect of *PMP22*, and affected by a variety of factors such as the dysregulation of the NRG1/ErbB pathway and the abnormality of lipid metabolism, etc. Current potential therapeutic strategies for CMT1A focus on correcting the gene dosage imbalance of *PMP22*. Among these, the most extensively studied drug, PXT3003, has completed Phase III clinical trials, demonstrating significant improvements in clinical symptoms and neuropathy scores. However, over-suppression of *PMP22* may reduce the dose from three copies to a single copy, thereby inducing the HNPP phenotype. Therefore, ideal treatment would require precise regulation of *PMP22* expression levels into the physiological range (two allele expression levels) rather than simply silencing the overexpressed gene.

Gene silencing techniques such as RNAi, CRISPR/Cas9 and ASOs have shown promising efficacy in animal models, but these approaches have not yet entered clinical trials, their long-term efficacy and safety remain unproven, and their dependence on viral vectors, potentially serious side effects, and off-target effects limit their direct clinical application. The development of induced pluripotent stem cell technology has brought us new hope in treating patients with CMT1A.

In conclusion, the pathogenesis of CMT1A is complex and drugs targeting different mechanisms are in the developmental stage. The combination of orthopedic, rehabilitative, surgical, and psychotherapeutic treatments, along with molecularly targeted therapies and stem cell replacement therapy, is expected to guide clinicians in the treatment of CMT1A, and to further improve patients’ neuromuscular function and quality of life.

## Supplementary Information

Below is the link to the electronic supplementary material.

Supplementary Material 1

## Data Availability

No datasets were generated or analysed during the current study.
