# Supplementary material for: The current status of Charcot-Marie-Tooth disease type 1 A treatment
Source: Acta Neurol Belg. 2025 Aug 27;125(6):1525–33. doi: 10.1007/s13760-025-02881-1 (PMC12644184; doi:10.1007/s13760-025-02881-1)
Supplement: Supplementary file 1 — Supplementary Material 1 [file 13760_2025_2881_MOESM1_ESM.docx]

**The Current Status of Charcot-Marie-Tooth Disease Type 1A Treatment**

**Article type**

Review article

**Title**

The Current Status of Charcot-Marie-Tooth Disease Type 1A Treatment

**Funding and Acknowledgments**

The study was supported by General Program of Jiangsu Natural Science Foundation (BK20241732), Jiangsu Postgraduate Practice and Innovation Program (SJCX24_0797) and Jiangsu Women and Children's Welfare Foundation. The authors express their sincere gratitude to the above-mentioned foundations for their generous support of this work.

**Author information**

***Authors and Affiliations***

**Department of Neurology, Children’s Hospital of Nanjing Medical University, Nanjing, 210019, China**

Hongdan Qi, Xin Wang, Bing Wu, Jing Chen and Gang Zhang

***Contributions***

**Hongdan Qi**: Conceptualization, Visualization, Writing – original draft, Writing – review & editing. **Xin Wang**: Investigation, Writing – original draft. **Bing Wu**: Writing – review & editing. **Jing chen**: Conceptualization, Supervision. **Gang Zhang**: Conceptualization, Supervision.

***Corresponding authors***

Jing Chen and Gang Zhang

E-mail addresses: dr.chenj@njmu.edu.cn and [zhanggangnjmu@126.com](mailto:zhanggangnjmu@126.com)

**Abstract**

Charcot-Marie-Tooth disease type 1A (CMT1A) is the major subtype of hereditary peripheral neuropathies and arises from a 1.5 megabase (Mb) tandem duplication in chromosome 17p11.2-p12 that contains the complete peripheral myelin protein 22 (*PMP22*) gene. Patients commonly present with progressive weakness and atrophy of the distal muscles, accompanied by hyperalgesia, decreased or absent tendon reflexes, and foot deformities. Current clinical management relies on multidisciplinary supportive care. Recent preclinical studies targeting potential therapeutic strategies for CMT1A have focused on correcting the gene-dose imbalance of *PMP22*. Notably, PXT3003 has shown phase III clinical efficacy in relieving symptoms and reducing neuropathy, and is expected to be the earliest CMT1A-targeted drug on the market. Gene editing approaches have also shown therapeutic promise in animal models, but off-target effects remain a concern. In addition, the rapid development of induced pluripotent stem cell (iPSC) technology has paved the way for stem cell therapies, which may be a promising therapeutic approach. This article reviews the existing literature on therapeutic strategies for CMT1A and aims to provide a valuable reference for the clinical treatment of CMT1A.

**Keywords**

Charcot-Marie-Tooth disease; CMT1A; *PMP22*; gene therapy; stem cell therapy; hereditary peripheral neuropathy

**Ethics Declarations**

***Competing interests***

The authors declare that there is no conflict of interest.

***Ethical Approval***

Not applicable.

***Consent for publication***

Not applicable.

***Availability of supporting data***

Not applicable.
